# Exploring Nutritional Properties, Bioactive Compounds, and Potential Applications of *Tamarindus indica* L.: An Underutilized Food Plant

**DOI:** 10.3390/foods15111953

**Published:** 2026-06-01

**Authors:** Yujiao Zhang, Ruimin Long, Chaohai Li, Lei Cheng, Rong Liu, Xi Liu, Baozhong Duan

**Affiliations:** 1 College of Pharmaceutical Science, Dali University, Dali 671000, China; 2 International Joint Laboratory for Research and Development of Yunnan’s Characteristic Medicinal and Edible Resources, Dali 671000, China

**Keywords:** *Tamarindus indica* L., nutritional composition, phytochemicals, biological activities, applications, safety evaluation

## Abstract

*Tamarindus indica* L. (tamarind) is a traditionally consumed food and medicinal plant with increasing relevance in the development of functional foods and bioactive natural ingredients. While the fruit pulp has been extensively utilized in food products, other fractions, including seeds, shells, and leaves, remain comparatively underexploited despite emerging evidence of notable nutritional and phytochemical value. This review summarizes recent progress regarding the nutritional composition, phytochemical characteristics, biological activities, safety considerations, and industrial applications of different parts of tamarind. These studies indicate that tamarind is rich in carbohydrates, dietary fiber, proteins, minerals, vitamins, polysaccharides, and phenolic compounds, which are associated with anti-oxidant, antihyperglycemic, hypolipidemic, anti-microbial, anti-inflammatory, and prebiotic effects. Nevertheless, most evidence is derived from in vitro and animal studies, while human clinical data remain scarce. In addition to their biological activities, tamarind-derived materials have shown promise in food formulation, pharmaceutical excipients, packaging systems, and environmental applications. Although these advances have been achieved, several challenges remain in compositional standardization, extraction efficiency, safety assessment, and clinical validation. Therefore, future research should focus on establishing standardized methods, optimizing extraction processes, improving safety evaluation systems, and conducting rigorous clinical trials to support the sustainable utilization of tamarind resources. Overall, this review provides a comprehensive scientific basis for the value-added development and sustainable utilization of tamarind resources.

## 1. Introduction

**Figure 1:**
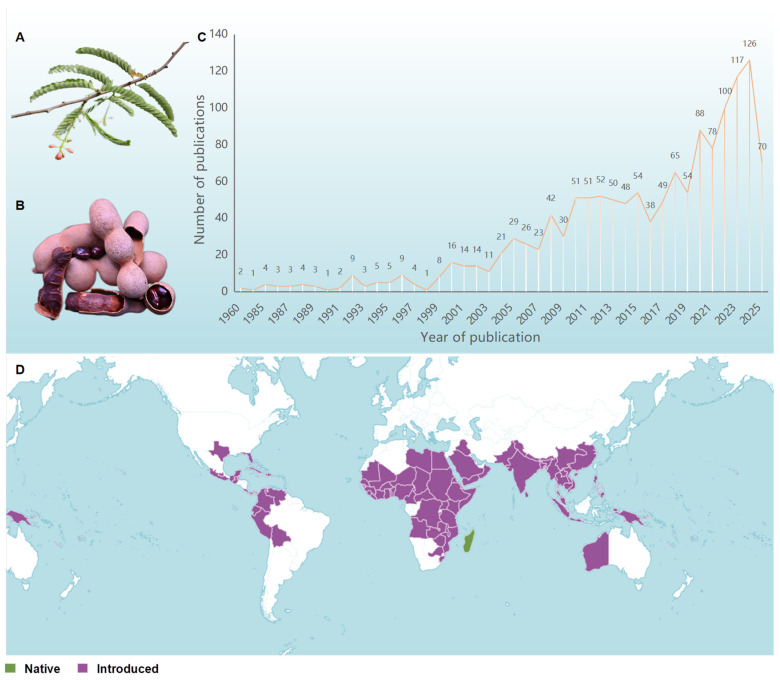
The morphological characteristics of tamarind (**A**,**B**), the tendency in the number of publications relevant to tamarind (1960–2025) (**C**), and the distribution diagram worldwide of tamarind (https://powo.science.kew.org/ (accessed on 15 February 2026)) (**D**).

*Tamarindus indica* L. (tamarind), also known as “suan jiao” or “luo wang zi” in China, belongs to the Fabaceae family and is widely cultivated throughout tropical and subtropical regions, particularly in India, Thailand, and southern China (Figure 1) [1,2,3]. The geographic distribution data were obtained from https://powo.science.kew.org/ (accessed on 15 February 2026). Tamarind has been used for centuries as both an herbal remedy and a dietary supplement. In traditional Chinese medicine, tamarind was recorded in the Materia Medica (*Ben Cao Gang Mu*, 1578), where it was described as having a sweet–sour flavor and cooling properties. It was traditionally used to relieve heat-related discomfort, improve digestion, and promote bowel movement [4]. Reflecting its historical use and well-established safety profile, tamarind was officially classified as a “functional food” by the National Health Commission of the People’s Republic of China in 2002, highlighting its integration into both traditional and modern healthcare practices.

The fruit pulp is the principal edible fraction of tamarind and accounts for approximately 40.9% of fresh fruit weight, which is highly valued due to its pleasant flavor and notable contents of proteins, vitamins, organic acids, and essential minerals [5]. In India alone, around 250,000 metric tons of tamarind fruit pulp were sold annually. In contrast, other parts, such as seeds, shells, and leaves, were often discarded as agro-industrial waste, despite evidence supporting their nutritional potential and bioactivity [6,7]. As of May 2025, more than 1384 tamarind-related publications have been indexed in the Web of Science (WOS) and China National Knowledge Infrastructure (CNKI) databases, with research dating back to 1960 (Figure 1B). Existing studies have mainly focused on extraction technologies, phytochemical characterization, and biological evaluation. Although several reviews have summarized specific aspects, such as fruit pulp composition, seed utilization, and health benefits [8,9], an integrated and up-to-date synthesis covering the whole plant remains lacking. Accordingly, a comprehensive overview is urgently needed to guide future research and utilization of tamarind resources. Previous reviews have primarily focused on the utilization of fruit pulp or seed polysaccharides [9,10]. In contrast, systematic comparisons among different plant fractions and critical evaluations of the strength of existing evidence remain limited. Moreover, current studies are highly fragmented, and industrial applications are often developed independently rather than within an integrated framework. These limitations hinder the rational and holistic valorization of tamarind as a whole-plant resource.

This study aims to provide a comprehensive, up-to-date review of tamarind research. A bibliometric analysis was conducted to identify research trends and assess global interest in tamarind. The nutritional components, phytochemicals, biological activities, safety aspects, and applications of tamarind were systematically summarized. Furthermore, the study highlights the challenges of tamarind in food and pharmaceutical products. Collectively, this review provides a scientific foundation and strategic insights for promoting the multi-functional use of tamarind.

## 2. Bibliometric Analysis

In the present study, bibliometric methods were systematically used to examine global research progress on tamarind. Relevant literature was retrieved up to 22 May 2025. The search strategy used the terms “*Tamarindus indica* L.” OR “*Tamarindus indica*” OR “tamarind” OR “*T. indica*” in the WOS database. In addition, both English and Chinese search terms were applied in CNKI, including “*Tamarindus indica* L.” OR “*Tamarindus indica*” OR “tamarind” OR “*T. indica*” or “酸角” or “罗望子” or “甜角”. Eligible records included peer-reviewed articles, review papers, conference proceedings, and academic theses that primarily addressed tamarind biology, phytochemistry, food applications, pharmacological properties, agricultural cultivation, environmental uses, or biomaterial development. Excluded records comprised duplicate entries, incomplete records lacking titles, abstracts, or keywords, non-scholarly materials (e.g., news reports or notices), and publications in which tamarind was only incidentally mentioned without substantive relevance.

**Figure 2:**
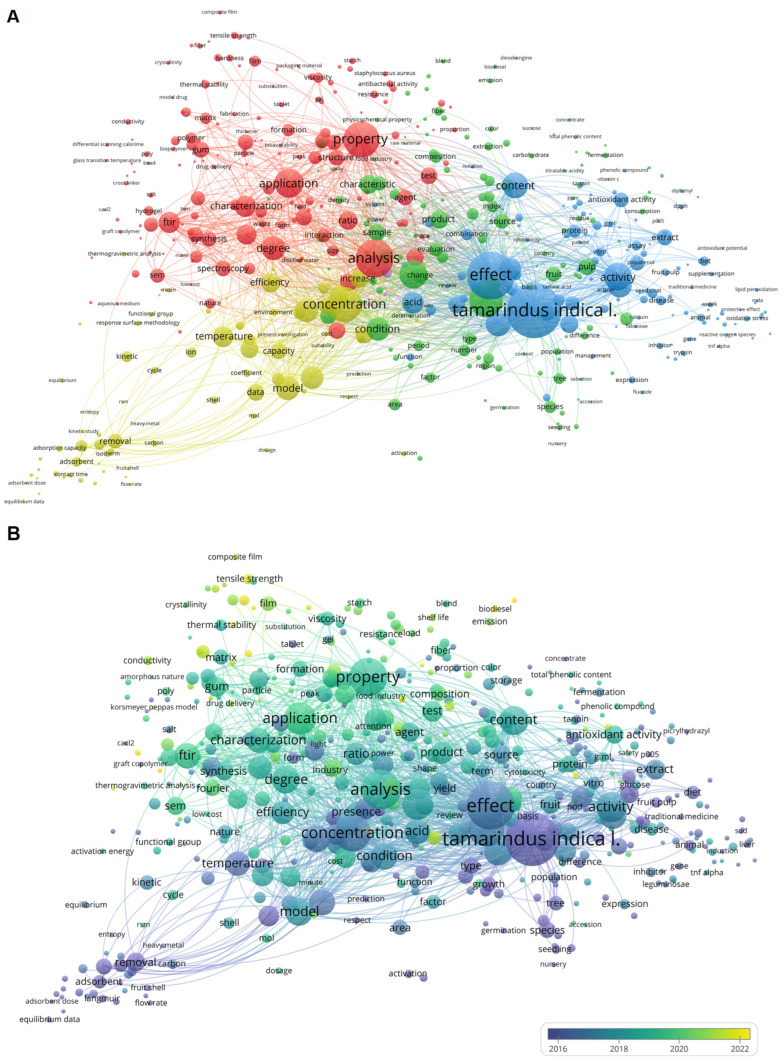
Keywords co-occurrence analysis of tamarind publications (**A**), and keywords analysis of tamarind publications with time information (**B**).

All retrieved records were manually screened based on titles and abstracts, and uncertain cases were further evaluated through full-text review. Following screening and data standardization, the final dataset consisted of 1116 records from WOS and 278 records from CNKI and was used for subsequent bibliometric analysis. Complete bibliographic information and cited references were exported in compatible formats and analyzed using VOS viewer. Keywords were primarily extracted from titles, abstracts, and author-provided keywords. In addition, synonymous terms, spelling inconsistencies, and singular/plural variants were unified to improve analytical accuracy. High-frequency keywords were then used to generate co-occurrence networks, thematic clusters, and temporal overlay maps. As shown in Figure 2, node size indicates the frequency of keyword occurrence, whereas line thickness reflects the strength of co-occurrence relationships between terms.

The keyword co-occurrence network analysis of tamarind literature revealed four distinct thematic clusters (Figure 2A), demonstrating the multidisciplinary nature of tamarind research. The first cluster (red) focuses on developing and characterizing tamarind-derived biomaterials, including composite films, hydrogels, and functional polymers, to enhance mechanical strength, thermal stability, and physicochemical properties for applications in packaging and drug delivery. The second cluster (blue) explores bioactive compounds from the fruit pulp, seed coat, and seeds, emphasizing their anti-oxidant and anti-microbial activities and investigating their potential use in functional foods and natural therapeutics. The third cluster (yellow) investigates the tamarind-based adsorbents for environmental remediation, focusing on removing heavy metals and organic pollutants using kinetic and isothermal models to assess adsorption capacity, efficiency, and regeneration potential. The fourth cluster (green) investigates the agronomic characteristics and nutritional composition of tamarind, providing insights into germplasm evaluation and sustainable utilization.

The timeline visualization of keyword co-occurrence further demonstrated a progressive shift in research priorities (Figure 2B). Early studies mainly focused on nutritional composition and biosorption potential. Subsequently, research attention moved toward the extraction and characterization of bioactive compounds, particularly anti-oxidants. In recent years, research interest has increasingly shifted toward advanced tamarind-based biomaterials, such as films, fibers, and hydrogels with enhanced structural and thermal properties. Meanwhile, emerging topics, including chemical modification, graft copolymerization, and biodiesel production, indicate expanding applications in sustainable materials science. Overall, tamarind research has progressed from basic compositional studies to diversified high-value applications in food science, pharmaceuticals, environmental engineering, and industrial technology.

## 3. Nutrients and Phytochemicals

**Figure 3:**
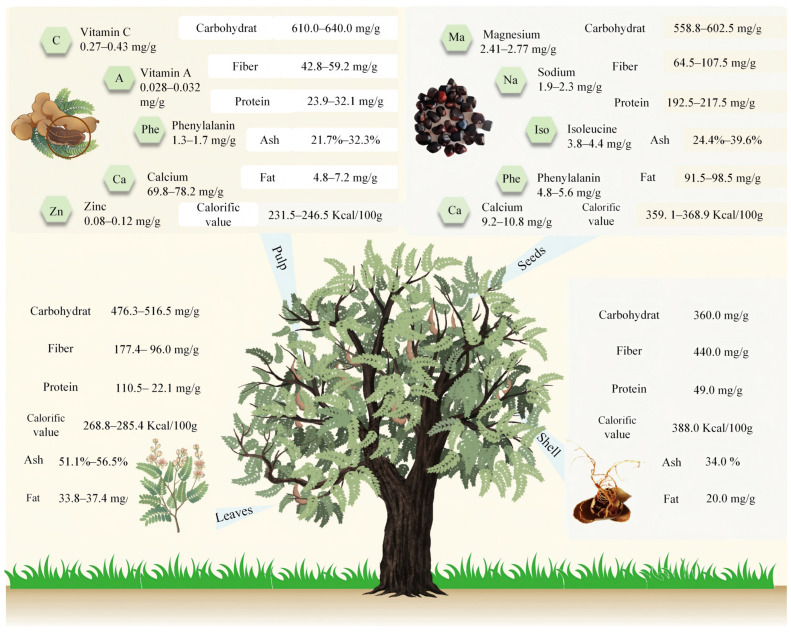
Nutritional components of different parts of tamarind.

Tamarind is recognized as a nutritionally valuable plant that contains a composition including carbohydrates, proteins, lipids, minerals, vitamins, and phytochemicals. Notably, the nutrient and phytochemical contents vary significantly among plant parts (Figure 3 and Table 1).

### 3.1. Carbohydrates

Tamarind is particularly valued for its high carbohydrates and dietary fiber content, which have significant health implications [21]. Tamarind fruit pulp generally contains the highest carbohydrate content. However, reported values vary depending on cultivar, maturity stage, moisture basis, and analytical methodology, which contains the highest carbohydrate content 610.0–640.0 mg/g DW [11], exceeding that of seeds (558.8–602.5 mg/g DW) [22], leaves (476.3–516.5 mg/g DW) [14], and shell (360.0 mg/g DW) [13]. This nutritional profile highlights the fruit pulp as the most carbohydrate-rich component of tamarind. In addition to carbohydrates, tamarind is a notable source of dietary fiber. For instance, the seeds contain 64.5–107.5 mg/g DW of dietary fiber [23,24,25] while the shell provides 440.0 mg/g DW [13]. Notably, the dietary fiber content in the shell is 1.8 times higher than that of roselle seed powder and 1.2 times higher than olive waste [26,27]. These findings suggest that tamarind, especially its fruit pulp and shell, is a valuable dietary component for increasing carbohydrate and fiber intake.

### 3.2. Proteins

Tamarind protein is increasingly recognized as a promising yet underutilized plant-based resource, offering environmental sustainability and higher nutritional value than conventional animal proteins [28]. Among its various parts, the seeds are particularly protein-rich, delivering 192.5–217.5 mg/g DW, significantly higher than wheat’s 130.0 mg/g DW [29]. Other parts, including the fruit pulp (23.9–32.1 mg/g DW), shell (49.0 mg/g DW), and leaves (110.5–122.1 mg/g DW), also contribute meaningful protein levels [11,13,14,22]. Beyond total protein content, tamarind seeds are particularly rich in essential amino acids critical for human nutrition, including isoleucine (3.8–4.4 mg/g DW), leucine (9.2–10.2 mg/g DW), and lysine (11.9–13.1 mg/g DW) [30,31,32]. These attributes make tamarind a valuable candidate for functional food applications, especially in regions facing malnutrition and limited access to animal-derived proteins.

### 3.3. Lipids

Lipids are found in trace amounts in tamarind shell, leaves, and fruit pulp, but are more concentrated in the seeds (Table 1). Specifically, tamarind fruit pulp contains relatively low lipid levels of 0.27% to 0.29%, while seeds contain significantly higher levels of 2.64% to 2.98% [22]. Tamarind seed oil is particularly notable for its high proportion of unsaturated fatty acids (55.6%) compared to saturated fatty acids (44.4%), a profile associated with cardiovascular and metabolic health benefits [33,34]. In addition to fatty acid composition, tamarind lipids also facilitate the absorption and transport of fat-soluble vitamins (A, D, E, and K) and carotenoids, which play crucial roles in immune function and cellular development [35,36,37]. Given these nutritional advantages, tamarind holds promise as a functional food with both dietary and therapeutic value.

### 3.4. Minerals

Tamarind is a source of essential minerals that play vital roles in human health. Its mineral composition varies among different plant parts. The fruit pulp, the most widely consumed portion, is high in potassium 596.6–659.4 mg/g DW, calcium (69.8–78.2 mg/g DW), and iron (26.5–29.5 mg/g DW), which collectively support electrolyte balance, bone mineralization, and red blood cell formation [38,39]. In contrast, the seeds are rich in sodium (1.9–2.3 mg/g DW), copper (0.80–0.92 mg/g DW), and zinc (0.9–1.1 mg/g DW), making them beneficial for maintaining bone integrity and managing hematological conditions [20,32].

Several factors also influence tamarind’s mineral content. The mineral concentration ranges from 2.20% to 2.40% in dry matter and 4.60% to 5.00% in fresh samples [5]. Seeds’ maturity affects mineral levels; for example, lighter-colored seeds contain more minerals (2.5 g/100 g) than darker seeds (2.17 g/100 g), an increase of approximately 15.00% [40]. Geographical and environmental conditions also impact mineral composition. Significant differences (*p* ≤ 0.005) were observed in the mineral profiles of tamarind fruit pulp and seeds collected from three agro-ecological zones in Uganda: Lake Victoria Crescent, Eastern, and West Nile regions [5]. In conclusion, tamarind is a mineral-rich plant that provides essential nutrients, supporting physiological functions critical to maintaining overall health.

### 3.5. Vitamins

Tamarind is recognized as a valuable source of essential vitamins, although current evidence is largely confined to analyses of the fruit pulp. As shown in Table 1, the pulp contains notable concentrations of vitamin C (0.27–0.43 mg/g DW), which exceed those of vitamin E (0.09–0.11 mg/g DW) and vitamin K (0.026–0.030 mg/g DW), and are comparable to or slightly higher than vitamin A levels (0.028–0.032 mg/g FW) [11]. In addition, the pulp is particularly rich in B-complex vitamins, including thiamine (0.40–0.45 mg/g DW), niacin (1.85–2.02 mg/g DW), and pyridoxine (0.061–0.071 mg/g DW) [11]. indicating its potential nutritional significance in dietary applications.

In contrast, data on the vitamin composition of other tamarind tissues, such as seeds, shells, and leaves, remain limited or largely unavailable in the current dataset. This data gap restricts comprehensive cross-tissue comparisons and hampers a holistic understanding of nutrient distribution within the plant. Therefore, further systematic and targeted investigations are warranted to establish a complete and reliable vitamin profile of tamarind across different anatomical parts.

### 3.6. Polysaccharides

**Figure 4:**
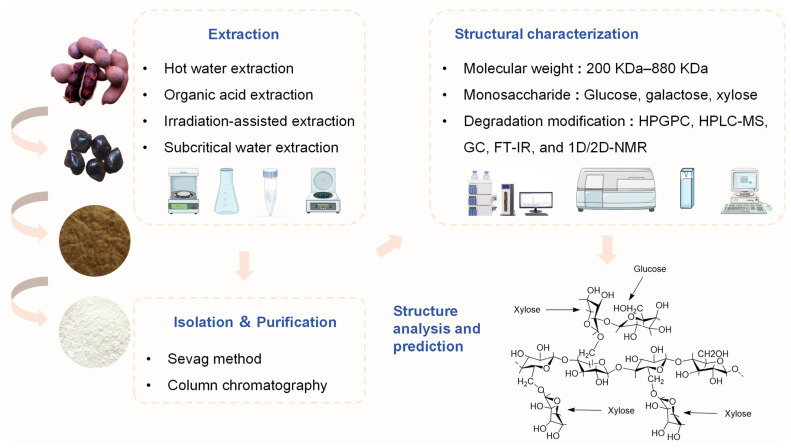
Schematic representation of the extraction, purification, and structure elucidation of tamarind seed polysaccharides.

Tamarind seed polysaccharides (TSP) are particularly notable for their functional versatility, which accounts for the highest percentage of tamarind seeds (approximately 50–60%) [41,42]. Recent advancements in extraction, purification, characterization, and modification techniques have accelerated research into their bioactivities (Figure 4). Notably, TSP is tightly bound within cell walls, necessitating extraction methods that balance efficiency with structural preservation. Common approaches include hot water extraction, organic acid treatment, irradiation-assisted methods, and subcritical water extraction, with ethanol precipitation often used for polysaccharide recovery post-extraction [43,44,45].

Variations in extraction methods lead to substantial differences in TSP molecular weight (*M*_w_). Reported values range from 196.1 kDa (TSP55 extracted at 55 °C) to 1735 kDa (via high-performance size-exclusion chromatography) [46,47,48], highlighting the sensitivity of *M*_w_ to methodological factors. Moreover, monosaccharide composition analysis, typically conducted via GC-MS or HPLC-MS, reveals glucose (45.09%), galactose (22.80%), and xylose (28.89%) as primary components [49], a profile critical to TSP’s functional behavior.

The structure of TSP largely determines its properties and biological activities, and it is therefore necessary to elucidate its structure using a combination of instrumental techniques. Advanced analytical techniques, including methylation analysis, FT-IR, and 1D/2D-NMR, have elucidated a (1 → 4)-β-D-glucan backbone with (1 → 6)-linked side chains of α-D-xylopyranose and β-D-galactopyranosyl-(1 → 2)-α-D-xylopyranose [48,50]. This unique configuration underpins TSP’s rheological properties, including its ability to form gels comparable to those of xanthan and guar gums [51], making it valuable in food processing, pharmaceuticals, and cosmetics [52]. In conclusion, the unique structural and functional properties of TSP, combined with advancements in extraction and analysis, highlight their promising potential in industries such as food, pharmaceuticals, and cosmetics. Further optimization of extraction methods and exploration of their bioactivity will expand their industrial applications.

### 3.7. Phenolic Compounds

Phenolic compounds possess diverse bioactive properties, including anti-oxidant, anti-diabetic, anti-microbial, and anticancer activities [53,54]. In tamarind, their distribution varies by tissue type, with the fruit pulp showing the highest total phenolic content (TPC) at 26.1–30.3 mg/g DW DW [11,55], followed by the seeds (60.2–70.6 mg/g DW), and leaves (21.3–28.1 mg/g DW) [13,14,56]. Extraction methods significantly affect phenolic yields. For example, microwave-assisted extraction enhances TPC in the shell by 1.3-fold [13], likely due to improved disruption of phenolic-matrix interactions [57]. Similarly, methanol extraction at 60 °C produces a higher seeds TPC than extractions at 20–40 °C, as elevated temperatures increase solvent penetration and promote cell wall breakdown [58]. The increased thermal energy enhances solvent penetration and facilitates cell wall degradation [59]. Notably, tamarind seeds contain higher concentrations of specific phenolic compounds compared to the fruit pulp. Key phenolics include procyanidin B2 (3.34–3.84 mg/g DW), procyanidin tetramer (18.4–21.1 mg/g DW), (–)-epicatechin (2.93–3.37 mg/g DW), procyanidin pentamer (10.7–12.3 mg/g DW), and procyanidin hexamer (14.5–16.7 mg/g DW) [11,18].

### 3.8. Other Compounds

The tamarind fruit pulp also contains volatile compounds, with aldehydes (26.38% to 31.82%), esters (18.81% to 23.12%), acids (9.27% to 12.03%), and alkenes (1.05% to 1.35%) as the predominant components [60]. In addition, analysis of tamarind leaves using UHPLC-ESI-MS revealed a diverse range of phytochemicals, including alkaloids, amides, esters, aldehydes, sesquiterpenes, steroids, and triterpenoids [61].

Overall, the chemical composition of tamarind varies considerably among its different parts. The fruit pulp is a major source of carbohydrates and phenolic compounds, whereas the seeds contain concentrated proteins, lipids, and polysaccharides. In addition, the leaves and shells provide valuable minerals, vitamins, and dietary fiber. Collectively, these findings suggest that tamarind should be recognized not only as a traditional fruit crop but also as a promising multi-functional resource, with potential applications in functional foods, nutraceuticals, and the development of sustainable ingredients.

## 4. Biological Activities

**Figure 5:**
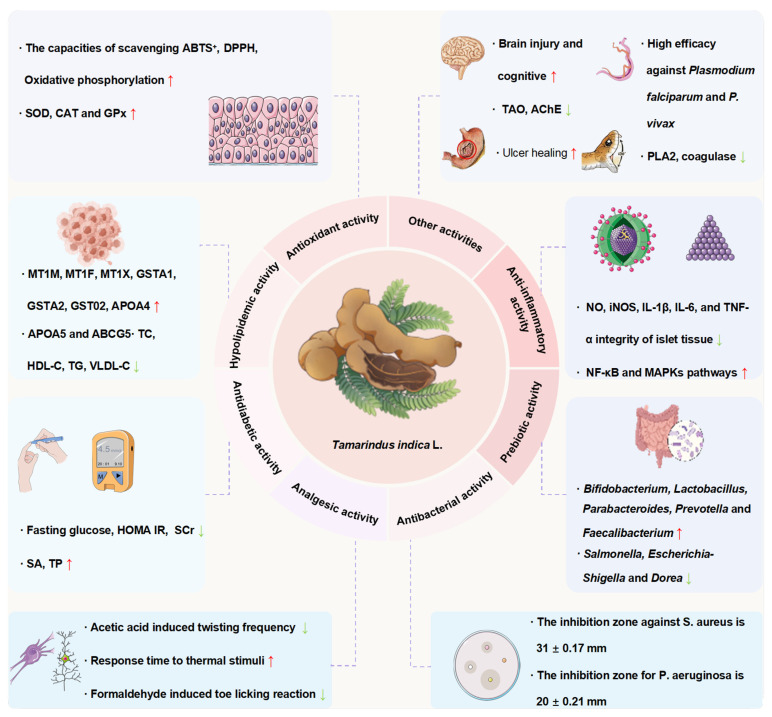
Main pharmacological activities of tamarind and their corresponding mechanisms of action. (“↓” decrease, “↑” increase).

Different parts of tamarind exhibit a wide range of reported biological activities, including anti-oxidant, hypolipidemic, antihyperglycemic, analgesic, antibacterial, prebiotic, and anti-inflammatory effects. However, the strength of evidence varies considerably across endpoints. The currently available evidence is summarized in Figure 5 and Table 2.

### 4.1. Antioxidant Activity

Tamarind-derived extracts and fractions have demonstrated anti-oxidant potential in both chemical and biological models; however, the magnitude of this activity varies depending on the plant fraction, extraction solvent, and assay system [114]. Its antioxidative effects have been extensively investigated in vitro using assays such as the 2,2-diphenyl-1-picrylhydrazyl (DPPH), 2,2′-azinobis(3-ethylbenzthiazoline-6-sulfonic acid) (ABTS), and ferric reducing anti-oxidant power (FRAP) assays [115]. Among tamarind-derived compounds, polysaccharides from fruit pulp exhibit more vigorous DPPH radical scavenging activity than those from seeds. Notably, microwave-pretreated tamarind shell displays higher DPPH activity than samples processed by ultrasound or maceration, supporting previous observations [13,116]. Methanol extracts from tamarind seeds and shell demonstrate significantly greater DPPH and ABTS radical scavenging activity than pomegranate and lychee [117]. The anti-oxidant activity of tamarind fruit pulp increases dose-dependently, as evidenced by elevated DPPH and FRAP activity at higher concentrations [63].

In addition to its in vitro efficacy, in vivo studies also demonstrated tamarind’s anti-oxidant properties. For example, treatment with tamarind shell extract (50–200 μg/mL) resulted in a dose-dependent enhancement of superoxide dismutase (SOD) activity (18% to 37%) and a reduction in malondialdehyde levels (22% to 41%) in a t-BHP-induced zebrafish [64]. Similarly, oral administration of tamarind seed coat extract (200 mg/kg) elevated the activities of SOD and catalase in CCl_4_-induced albino rats [118]. Moreover, a combined formulation containing hesperidin, crocetin, and tamarind extract effectively alleviated N-methyl-D-aspartate-induced retinal cell apoptosis in a mouse model of oxidative stress [119].

### 4.2. Hypolipidemic Activity

In recent years, abnormal lipid metabolism has become a primary global health concern, contributing to obesity and dyslipidemia [120]. Multiple animal studies have shown that tamarind-derived extracts may lower lipid levels, particularly in models of diet-induced obesity and dyslipidemia. For instance, administering a tamarind seed trypsin inhibitor (TTI) at 730 µg/kg/day for 10 days reduced food intake. It lowered triglyceride and very low-density lipoprotein cholesterol levels in obese Wistar rats [68]. Similarly, oral administration of tamarind fruit pulp extract (50–100 mg/kg for 40 days) significantly decreased serum total cholesterol and triglycerides in sulpiride-induced obese mice, while increasing high-density lipoprotein cholesterol (HDL-c) levels [121]. These effects correlated with upregulation of Abcg5, Cyp1A1, and Gstm1; downregulation of HMG-CoA reductase and Mtp; and inhibition of LDL receptor expression [122]. Additionally, tamarind seed extract showed greater antihyperlipidemic effects than fruit pulp or leaf extracts in a lard-induced obesity rat model [67]. Moreover, seed extract inhibited adipogenesis, reducing intracellular lipid accumulation and blocking fatty acid conversion at 1.25–10 µg/mL [69]. Separately, tamarind leaves extract showed potent pancreatic lipase inhibition with an adequate concentration of 3.8 μg/mL [61].

### 4.3. Antidiabetic Activity

Diabetes mellitus is a chronic disease characterized by high morbidity and mortality, posing a serious threat to human health. Recent studies have shown that tamarind has anti-diabetic potential through enzyme inhibition and in diabetic rodent models, both in vivo and in vitro. For instance, oral administration of TTI derived from methanol extracts of tamarind seeds significantly reduced fasting blood glucose (FBG) and improved insulin sensitivity, as indicated by decreased homeostatic model assessment of insulin resistance values and increased HDL-c levels in diet-induced type 2 diabetic rats [73]. In the same model, nanoencapsulation of TTI using ethyl cellulose as the wall material significantly enhanced its hypoglycemic effect, likely due to controlled release in the gastric environment [74,75]. Additionally, ethanol extracts (200 mg/kg) of tamarind leaves significantly reduced FBG levels at both 8 and 12 h after administration in acute hyperglycemia models, compared to untreated diabetic controls [89].

In vitro studies further support tamarind’s anti-diabetic potential through multiple mechanisms. Bioactive constituents from tamarind leaves and fruit pulp have been found to strongly inhibit α-amylase and α-glucosidase, two key enzymes in carbohydrate digestion [77,123]. Additionally, molecular docking analyses revealed that tamarind leaf-derived compounds, such as linalool, anthranilate, and hexadecanol, can inhibit protein tyrosine phosphatase, an enzyme implicated in insulin resistance [78]. Collectively, these findings underscore tamarind’s promise as a candidate for the development of novel hypoglycemic agents or complementary therapies.

### 4.4. Analgesic Activity

Tamarind demonstrates notable analgesic effects through central and peripheral pathways [124]. Methanolic extracts of tamarind leaves exhibit dose-dependent pain relief (50–200 mg/kg), reducing acetic acid-induced writhing in mice by 33.6% to 47.2%. Moreover, offering thermal pain protection ranging from 17.8% to 64.9% [82]. Additionally, recent studies highlight the synergistic analgesic effects of combining tamarind-derived anthocyanins with curcumin from turmeric, particularly in relieving dysmenorrhea in young women. This combination significantly reduces menstrual pain by inhibiting uterine contractions and modulating inflammatory responses [81]. Clinical evidence further supports tamarind’s analgesic efficacy, demonstrating that seed extracts reduce pain in patients with mild to moderate knee osteoarthritis by exerting anti-inflammatory and anti-oxidant effects. Specifically, these extracts suppress pro-inflammatory markers such as tumor necrosis factor-alpha (TNF-α), Interleukin-6, high-sensitivity C-reactive protein, and Matrix Metalloproteinase-3, and decrease cartilage degradation markers such as urinary C-telopeptide of type II collagen [80]. Collectively, these findings highlight the therapeutic potential of tamarind for pain management and support its development as a natural analgesic.

### 4.5. Antibacterial Activity

Microbial infections remain a primary global health concern [125]. Recent studies have demonstrated that acetone and ethanolic extracts of tamarind fruit pulp exhibit potent antibacterial activity against both Gram-positive bacteria, such as *Staphylococcus aureus*, and Gram-negative pathogens, including *Pseudomonas aeruginosa* and *Klebsiella pneumoniae* [85,87]. Ethanolic extracts from tamarind leaves also show notable bacteriostatic effects, producing inhibition zones of 20 mm against *Escherichia coli* and 17 mm against *Salmonella* spp. at 100 mg/mL [88]. In addition to these extracts, tamarind-derived structural materials also demonstrated promising anti-microbial potential. For example, composite films made by xyloglucan extracted from tamarind seed kernel-derived xyloglucan and chitosan (in ratios from 1:1 to 4:1) could effectively suppress microbial growth, maintaining microbial counts below 30 CFU/g, which is attributed to synergistic chemical interactions between xyloglucan and chitosan, as well as modifications to the films’ physical structure [84].

### 4.6. Prebiotic Activity

TSP demonstrates dose-dependent prebiotic activity by enhancing probiotic growth, suppressing pathogenic bacteria, and modulating microbial metabolites. A recent study reported that tamarind seed kernel powder promoted the growth of *Bifidobacterium animalis* at 22%·h^−1^ and significantly enhanced biofilm formation (BFI = 256.71), outperforming inulin at 2.5% to 5.0% [90]. Notably, two polysaccharide fractions—ETSP1 (176.68 kDa) and ETSP2 (34.34 kDa) stimulated the growth of beneficial gut bacteria such as *Bacteroides*, *Bifidobacterium*, *Parabacteroides*, and *Faecalibacterium*. This effect was associated with elevated production of short-chain fatty acids (SCFAs) and reduced levels of harmful bacteria, including *Escherichia*, *Shigella*, and *Dorea*, consistent with earlier research [92,93]. Furthermore, hot-water-extracted polysaccharides from tamarind seeds have been shown to promote probiotic growth, boost SCFA synthesis, and lower the pH of fermentation broth, thereby fostering a more favorable intestinal environment [94]. Collectively, these results highlight TSP as a promising prebiotic candidate for improving gut health.

### 4.7. Anti-Inflammatory Activity

Inflammation is a protective response of the immune system to injury or harmful stimuli, mediated by cytokines that regulate the immune cascade [126]. Recent studies have shown that tamarind extracts possess notable anti-inflammatory properties, particularly by alleviating osteoarthritic pain and joint inflammation induced by monosodium iodoacetate in mice. These effects are primarily mediated by inhibition of the NF-κB signaling pathway, leading to reduced expression of key pro-inflammatory mediators, including TNF-α, interleukin-1β, nitric oxide, inducible nitric oxide synthase, and cyclooxygenase-2 [98,127]. In addition, tamarind leaves extract could be formulated into a transferosomal gel, significantly reducing paw edema volume in carrageenan-induced rat models [65,97]. Moreover, tamarind leaves extract showed the most potent anti-inflammatory effect in LPS-stimulated RAW 264.7 macrophages, followed by seeds, fruit, and bark extracts [99]. Overall, these findings position tamarind as a potent natural anti-inflammatory agent with broad potential in managing chronic inflammatory disorders.

### 4.8. Other Activities

Tamarind extract exhibits diverse pharmacological properties, including neuroprotective, anti-ulcer, antimalarial, and other biological activities. Specifically, methanolic extracts from tamarind bark and seeds have been shown to inhibit acetylcholinesterase and butyrylcholinesterase, enzymes associated with neurodegenerative conditions [103]. Tamarind also demonstrates strong anti-ulcer properties. Methanolic extracts from the seeds coat protect against gastric lesions induced by ibuprofen, alcohol, and pyloric ligation in rats, while leaf extracts promote ulcer healing with repair rates exceeding 50% [105,128]. Moreover, fruit and leaf extracts exhibit potent antimalarial effects. Chloroform extracts show high efficacy against *Plasmodium falciparum* and *Plasmodium vivax*, with performance comparable to that of chloroquine [23,106]. Beyond these, tamarind seed extract has demonstrated antiviral activity against SARS-CoV-2 and *Ascaris suum* [107,112]. Furthermore, seed coat extracts exhibit antivenom effects through inhibiting phospholipase A2 and coagulase enzymes [110,112]. These findings remain preliminary, and the overall strength of evidence is considered weak.

Overall, tamarind exhibits diverse biological activities, including anti-oxidant, hypolipidemic, anti-diabetic, analgesic, antibacterial, prebiotic, and anti-inflammatory effects, as demonstrated in in vitro and animal studies. However, the overall strength of the evidence varies considerably. Most findings are derived from chemical assays or rodent models, with relatively few well-controlled clinical trials available. Moreover, variations in extraction methods, dosages, and endpoints make direct comparisons between studies difficult. The limited availability of human data, unclear underlying mechanisms, and insufficient evaluation of practical applications (e.g., stability and matrix interactions) represent major research gaps. Future studies should prioritize well-designed and standardized clinical studies, comprehensive structure–activity relationship analyses, and in-depth mechanistic investigations to confirm their translational potential. In addition, the development of tamarind-based functional products with scientifically validated health benefits represents a promising research direction.

## 5. Safety Concerns

Toxicity assessment is essential for determining the safety of natural products. Existing toxicological studies generally suggest a favorable safety profile for certain tamarind extracts. For example, a six-month study reported no significant changes in hematological or biochemical parameters following oral administration of tamarind fruit pulp water extract in rats [129]. Likewise, no acute toxicity was observed in rats given tamarind seed coat extract, even at 4000 mg/kg [76]. In a related study, a trypsin inhibitor isolated from tamarind seeds demonstrated no genotoxicity, systemic toxicity, and tissue damage in obese male rats at bioactive doses [130,131]. Similarly, tamarind leaf extract showed no hemolytic activity in vitro and no adverse effects in animal models at doses up to 5000 mg/kg [11]. Moreover, the cytotoxicity of tamarind shell extract (TSE) was evaluated on ATDC5 cells using the MTT assay. At concentrations below 250 μg/mL, TSE exhibited minimal cytotoxicity [64]. Overall, these findings suggest a relatively favorable safety profile for tamarind-derived materials. Nevertheless, the current evidence remains limited, largely due to short study durations, a heavy reliance on animal models, and the lack of comprehensive evaluations of chronic toxicity, reproductive toxicity, allergenicity, and potential herb–drug interactions. Consequently, the limited scope and depth of existing studies continue to hinder their clinical translation.

## 6. Applications

Growing recognition of tamarind’s nutritional value and health benefits has heightened interest in its unique functional and physiological properties. Consequently, demand for tamarind-based products is rising, driving the introduction of diverse value-added goods into global markets.

### 6.1. Food Formulation

Tamarind is widely used in food processing for its high nutritional value and distinctive sweet–sour flavor. Its fruit pulp is a key ingredient in products such as sauces, beverages, candies, and ice creams. It has been identified to contain 21 key aroma-active compounds, reinforcing its role as a natural flavor enhancer [132]. Additionally, TSP, when hydrolyzed with cellulase, exhibits low-fat characteristics, making it ideal for low-calorie food formulations [12]. TSP also inhibits ice recrystallization, suggesting its potential as an innovative antifreeze agent in frozen food applications [133]. Moreover, tamarind seed powder and seed coats, rich in natural anti-oxidants, are valuable functional additives that enhance food quality and shelf life [69,134].

### 6.2. Pharmaceuticals

Tamarind shows strong potential in the pharmaceutical industry as a natural polymer, owing to its low cost, non-toxicity, biocompatibility, biodegradability, and non-irritant properties. These features make it an excellent candidate for drug formulation. For instance, TSP functions as a suspending and emulsifying agent in liquid oral preparations, a binder in solid dosage forms, and a release modifier in controlled-release drug delivery systems [135,136,137]. Moreover, phytochemicals derived from tamarind seeds have been used as eco-friendly biocatalysts in the synthesis of magnesium oxide nanoparticles, which exhibit cardioprotective, antibacterial, and anti-oxidant properties—further supporting their biomedical relevance [55,138]. Furthermore, contemporary research has expanded the scope of tamarind’s medicinal applications, demonstrating its efficacy as a functional food and dietary supplement for diabetes management [139].

### 6.3. Other Fields

Beyond its pharmaceutical and food uses, tamarind also holds significant potential in materials science and sustainable technologies. For instance, gold/titanium dioxide nanocomposites synthesized from extracts of tamarind fruit pulp and Pilea melastomoides leaves have demonstrated vigorous photocatalytic activity in degrading the methylene blue dye in wastewater treatment [140]. Moreover, natural photosensitizers derived from Capsicum annuum and tamarind seed extracts have been successfully used to develop dye-sensitized solar cells [141].

In conclusion, the diverse bioactivities and physicochemical properties of tamarind and its derivatives highlight their broad potential for functional foods, medicinal formulations, and environmentally friendly industrial applications.

## 7. Conclusions and Perspectives

Accumulating evidence suggests that tamarind should be reconsidered not merely as a traditional souring fruit, but as a multi-functional bioresource with applications spanning nutrition, functional ingredients, pharmaceuticals, and sustainable materials. Studies have shown that tamarind is particularly rich in macronutrients, including proteins and dietary fiber, as well as micronutrients such as essential minerals (e.g., calcium, zinc, and iron) and vitamins (e.g., vitamins A and C). In addition, it contains a wide array of bioactive phytochemicals, including polysaccharides and phenolic compounds such as taxifolin and eriodictyol. These diverse chemical constituents contribute to tamarind’s broad spectrum of biological activities, including anti-oxidant, anti-diabetic, anti-inflammatory, anti-microbial, and nephroprotective effects. Preclinical studies have demonstrated low toxicity and high therapeutic efficacy, supporting its potential in health supplements and chronic disease management.

In addition to its nutritional and pharmacological applications, tamarind has shown promise across multiple domains. TSPs serve as natural binders and controlled-release agents in pharmaceutical formulations. The fruit pulp is also frequently incorporated into traditional Chinese medicinal recipes and into various culinary products, such as beverages, candies, and sauces. Despite these advancements, several research and development gaps remain:

(i) Underutilization of non-fruit pulp fractions: While tamarind fruit pulp has been widely studied and applied, other plant parts—including seeds, shells, and leaves—are often discarded as waste, despite their documented nutritional value and potential pharmacological properties. Systematic investigations of these fractions are necessary to exploit their potential fully.
(ii) Incomplete phytochemical characterization: Although many compounds have been isolated from tamarind, detailed structural identification and structure–activity relationship studies are still lacking. Advanced techniques such as LC-MS/MS, NMR, and metabolomic analysis are essential for comprehensive characterization.
(iii) Need for quality control and standardization: The commercialization of tamarind-based products in the food and pharmaceutical sectors requires rigorous quality assurance systems. Methods like chemical fingerprinting, bioactivity-guided profiling, and standardization of active components are crucial to ensure consistency, efficacy, and safety.
(iv) Expansion of industrial and biomedical applications: Due to their biocompatibility, safety, and multi-functional bioactivity, tamarind-derived substances—especially polysaccharides and polyphenols—are promising for the development of materials such as drug delivery platforms, biofilms, and nutraceuticals. However, issues related to formulation, stability, and large-scale production must be resolved to support practical applications.
(v) Limited toxicological and safety evaluation: Although tamarind is generally regarded as safe, the toxicological profiles of non-fruit pulp parts and their modified derivatives remain underexplored. There is an urgent need for standardized safety evaluations and pharmacokinetic studies to facilitate regulatory approval and public trust.
(vi) Extraction and processing bottlenecks: Current techniques for isolating tamarind phytochemicals are mostly limited to laboratory settings. To achieve industrial scalability, it is necessary to develop eco-friendly, cost-efficient, and high-yield extraction methods—such as membrane separation, ultrasound-assisted extraction, and integrated biorefinery strategies.

In conclusion, tamarind research offers both considerable opportunities and persistent challenges. This review summarizes current advances and provides insights that may guide future studies on tamarind and its related products. However, fully realizing its potential will require a shift from preliminary investigations toward application-oriented innovation. Future research should focus on the comprehensive utilization of the whole plant, the optimization of modern extraction and purification techniques, and the establishment of standardized quality control and safety evaluation frameworks [142]. In addition, greater emphasis on mechanistic studies and well-designed human clinical validation will be essential for future progress.

## Figures and Tables

**Table 1:** Nutrient values of different parts of tamarind.

Composition	Unit	Seeds	Fruit Pulp	Shell	Leaves	Composition
Proximate						
Moisture	mg/g FW	79.5–89.2	302.0–328.0	98.0	105.6–116.8	[11,12,13,14]
Carbohydrats	mg/g DW	558.8–602.5	610.0–640.0	360.0	476.3–516.5	[11,12,13,15]
Fiber	mg/g DW	64.5–107.5	42.8–59.2	440.0	177.4–196.0	[11,12,13,15]
Protein	mg/g DW	192.5–217.5	23.9–32.1	49.0	110.5–122.1	[11,12,13,16]
Fat	mg/g DW	91.5–98.5	4.8–7.2	20.0	33.8–37.4	[11,12,13,15]
Calorific value	Kcal/100 g DW	359.1–368.9	231.5–246.5	388.0	268.8–285.4	[11,12,13]
Ash	mg/g DW	24.4–39.6	21.7–32.3	34.0	51.1–56.5	[11,12,13,15]
Amino acids						
Isoleucine	mg/g DW	3.8–4.4	1.0–1.4	—	—	[15,16]
Leucine	mg/g DW	9.2–10.2	1.8–2.4	—	—	[15,16]
Lycine	mg/g DW	11.9–13.1	1.6–2.0	—	—	[15,16]
Methionine	mg/g DW	1.6–2.0	0.4–0.6	—	—	[15,16]
Cysteine	mg/g	1.0–1.2	0.2–0.4			[16]
Phenylalanin	mg/g DW	4.8–5.6	1.3–1.7	—	—	[15,16]
Tyrosine	mg/g DW	2.1–2.5	0.6–0.8	—	—	[16]
Threonine	mg/g DW	3.3–3.9	1.0–1.2	—	—	[15,16]
Valine	mg/g DW	4.2–4.8	1.1–1.5	—	—	[15,16]
Arginine	mg/g DW	17.1–19.3	2.3–2.9	—	—	[15,16]
Histidine	mg/g DW	2.7–3.1	0.5–0.7	—	—	[16]
Alanine	mg/g DW	7.3–8.3	2.0–2.4	—	—	[16]
Aspartic	mg/g DW	14.4–16.8	4.1–4.9	—	—	[16]
Glutamic	mg/g DW	26.4–30.6	6.3–7.3	—	—	[15,16]
Glycine	mg/g DW	6.1–6.9	1.7–2.1	—	—	[16]
Proline	mg/g DW	9.5–11.1	4.7–5.5	—	—	[16,17]
Serine	mg/g DW	5.4–6.2	1.5–1.9	—	—	[16]
Minerals						
Macro-element						
Magnesium	mg/g DW	16.8–19.2	86.9–97.1	—	—	[11,18]
Sodium	mg/g DW	1.9–2.3	2.5–3.1	—	—	[11,18]
Calcium	mg/g DW	9.2–10.8	69.8–78.2	—	—	[11,14,18]
Phosphorus	mg/g DW	23.7–27.3	106.5–119.5	—	—	[11,14,18]
Potassium	mg/g DW	62.5–71.5	596.6–659.4	—	—	[11,18]
Aluminum	mg/g DW	1.9–2.3	—	—	—	[11]
Micro-element						
Copper	mg/g DW	0.80–0.92	0.81–0.91	—	—	[11]
Iron	mg/g DW	71.7–80.1	26.5–29.5	—	—	[11]
Zinc	mg/g DW	0.9–1.1	0.08–0.12	—	—	[11,18]
Selenium	mg/g DW	0.011–0.015	0.011–0.015	—	—	[11]
Manganese	mg/g DW	2.41–2.77	—	—	—	[11]
Vitamin						
Niacin	mg/g DW	—	1.85–2.02	—	—	[11,19]
Pantothenic acid	mg/g DW	—	0.13–0.15	—	—	[11]
Pyridoxine	mg/g DW	—	0.061–0.071	—	—	[11]
Thiamin	mg/g DW	—	0.40–0.45	—	—	[11,19]
Vitamin A	mg/g FW	—	0.028–0.032	—	—	[11,19]
Vitamin C	mg/g DW	—	0.27–0.43	—	—	[11,12]
Vitamin E	mg/g DW	—	0.09–0.11	—	—	[11]
Vitamin K	mg/g DW	—	0.026–0.030	—	—	[11]
β-Carotene	mg/g DW	—	0.017–0.019	—	—	[11]
Fatty acids						
Total lipid	mg/g DW	91.5–98.5	4.8–7.2	—	—	[11,15]
Palmitic acid (C16:0)	% DW	11.9–14.1	—	—	—	[11,20]
Stearic acid (C18:0)	% DW	4.6–5.4	—	—	—	[11,20]
Oleic acid (C18:1)	% DW	7.2–8.4	—	—	—	[11,20]
Arachidic acid (C20:0)	% DW	0.8–1.0	—	—	—	[11,20]
Behenic acid (C22:0)	% DW	0.9–1.1	—	—	—	[11,20]
Lignoceric acid (C24:0)	% DW	1.4–1.6	—	—	—	[11,20]
Phenolic compounds						
Total Phenolic	mg/g DW	60.2–70.6	26.1–30.3	—	21.3–28.1	[11,13,18]
Total Flavonoids	mg/g DW	15.2–17.6	—	—	10.0–16.6	[11,13,18]
(+)-Catechin	mg/g DW	—	0.52–0.60	—	—	[11,18]
Procyanidin B2	mg/g DW	3.34–3.84	2.15–2.47	—	—	[11,18]
(−)-Epicatechin	mg/g DW	2.93–3.37	2.46–2.82	—	—	[11,18]
Procyanidin trimer	mg/g DW	11.0–12.6	2.95–3.39	—	—	[11]
Procyanidin tetramer	mg/g DW	18.4–21.1	5.82–6.70	—	—	[11]
Procyanidin pentamer	mg/g DW	10.7–12.3	3.03–3.49	—	—	[11]
Procyanidin hexamer	mg/g DW	14.5–16.7	3.35–3.85	—	—	[11]
Polymeric tannins	mg/g DW	217.6–250.4	127.1–146.7	—	—	[11]
Taxifolin	mg/g DW	—	1.94–2.24	—	—	[11]
Apigenin	mg/g DW	—	0.51–0.59	—	—	[11]
Eriodictyol	mg/g DW	—	1.80–2.06	—	—	[11]
Luteolin	mg/g DW	—	1.30–1.50	—	—	[11]
Naringenin	mg/g DW	—	0.37–0.43	—	—	[11]

Note: mg/g FW = milligrams per gram fresh weight; mg/g DW = milligrams per gram dry weight; % DW = percentage of total fatty acids on a dry weight basis; — = Data not found.

**Table 2:** Beneficial effects of different tamarind parts (“↓” decrease, “↑” increase).

Plant Part	Sample Form	Experiment	Major Results	References
Anti-oxidant activity				
Tamarind fruit pulp	Aqueous extract	In vitro (DPPH) and in vivo (anti-TB drugs induced rats)	The in vitro anti-oxidant activity is 81.48% SOD, CAT and GSH ↑	[62]
Tamarind fruit pulp	70% ethanol extracts	In vitro (DPPH and FRAP)	The anti-oxidant capacity is closely related to the total phenolic content DPPH, FRAP < gallic acid	[63]
Tamarind shell	95% ethanol reflux extraction	In vitro (ORAC) and in vivo (t-BHP induced zebrafish)	Antioxidant capacity: taxifolin > morin > (+)- epicatechin > naringin > eriodictyol > luteolin > myricetin > apigenin T-BHP induced oxidative stress ↓	[64]
Tamarind shell	60% ethano extracts	In vitro (DPPH, ABTS, and FRAP)	The anti-oxidant capacity was found to follow the order: microwave pretreatment > ultrasonic pretreatment > maceration	[13]
Tamarind leaves	Ethanol extract	In vitro (DPPH)	The IC_50_ values of ethanol and watery extracts of tamarind leaves were 34.74 and 57.13 μg mL^−1^	[14]
Tamarind Bark	99% ethanol extracts	In vitro (DPPH)	DPPH > Hydrogen peroxide The total anti-oxidant capacity and total phenolic content are similar	[65]
Tamarind root	Macerated using ethanol	In vitro (TAC)	TAC: 109.57 (mg VCE); TPC: 64.04 GAE 100 g^−1^	[66]
Hypolipidemic activity				
Tamarind seeds (TS), fruit pulp (TP), leaves (TL)	96% ethanol immersion for 3–5 days	In vivo (lard induction)	TC, HDL-C by TS and TL ↓ Ativity: TF > TS > TP	[67]
Tamarind seeds	Soak in 50 mM Tris-HCl buffer (pH 7.5)	In vivo (diet-induced rats)	TG, VLDL-C ↓ Food intake ↓	[68]
Tamarind seeds coat	The maceration technique	In vitro (3T3-L1 adipocytes)	Adipose cell differentiation and adipogenesis ↓ Within the concentration range of 2.5–10 µg/mL: TG ↓	[69]
Tamarind fruit pulp	10% aqueous fruit pulp extract	In vivo (male Sprague–Dawley rats)	TC, LDL, and triglycerides ↓ HDL ↑	[70]
Tamarind fruit pulp	Fruit juice	In vivo (male Wistar albino rats)	TC, TG, LDL, VLDL ↓ HDL ↑	[71]
Tamarind fruit pulp	Soaked in 50 mL of methanol for 24 h	In vitro (human HepG2 cells)	MT1M, MT1F, MT1X, GSTA1, GSTA2, GST02, APOA4, APOA5 and ABCG5 ↑ PDE3A, PTTG1, CYP1A1, IFIT1 and MTTP ↓ CCNG1, CCNG2, CCNA2, CCNB1, CCNB2 and CDC2 ↓	[72]
Anti-diabetic activity				
Tamarind seeds	Trypsin inhibitor	In vivo (diet-induced Wistar rats)	Fasting glucose, HOMA IR ↓ HDL-c ↑	[73,74]
Tamarind seeds	TTI	In vivo (Wistar rats)	The blood glucose levels ↓ Insulin resistance ↑	[75]
Tamarind seeds	70% ethanol extracts	In vivo (Wistar rats)	Glucose uptake ↑ Ttherapeutic effect < insulin	[76]
Tamarind seeds coat	From Synthite Industries Ltd.	In vitro (α-amylase and α-glucosidase)	α-amylase and α-glucosidase inhibitory activities ↑ The IC_50_ value for α-glucosidase inhibition was 34.19 µg/mL, which is comparable to that of the standard drug acarbose (IC_50_ = 34.83 µM)	[77]
Tamarind fruit pulp	Extract	In vitro (molecular docking)	Linalool antianilate, Hexadecanol, Pentadecanol, and Benzyl benzoate) showed potential antihypergly activity The anti diabetes activity < the known drug glimepiride	[78]
Tamarind leaves	Ethanol extract (TIME)	In vivo (STZ-induced diabetic rats)	Blood glucose levels at 8 and 12 h post-dosing ↓ No acute toxicity was observed with TIME at a dose of 2000 mg/kg body weight	
Tamarind leaves	Extracted in a Soxhlet extractor with ethanol	In vivo (Wistar rats)	SCr ↓ SA, TP ↑	[79]
Analgesic activity				
Tamarind seeds	TS and *Turmeric rhizome* ethanol extracts	In vivo (human clinical trials)	Pain in patients with mild to moderate knee osteoarthritis ↓	[80]
Tamarind fruit pulp	Tamarind-turmeric drink	In vivo (young women)	The average pain ↓ The analgesic effect is related to curcumin and anthocyanins	[81]
Tamarind leaves	70% ethanol extracts	In vivo (adult Swiss albino mice)	Acetic acid induced twisting frequency ↓ Response time to thermal stimuli ↑ Formaldehyde induced a toe-licking reaction ↓	[82]
Antibacterial activity				
Tamarind seeds	Ethyl acetate extraction	In vitro (paper disc diffusion method)	The diameter of the inhibition zone against *E. coli* is 7.90 mm	[83]
Tamarind seeds kernel	Extract the tamarind seed kernel	In vitro (total Viable Count Test)	When the ratio of xyloglucan to chitosan is 1:1, 2:1, 3:1, and 4:1, the microbial growth rate is below 30 CFU/g, indicating that the film has excellent antibacterial ability The antibacterial activity of chitosan can be enhanced by adding xyloglucan	[84]
Tamarind fruit pulp	80% methanol extraction	In vitro (16S rRNA gene detection and Kirby-Bauer disk diffusion)	The inhibition zone against *S. aureus* is 31 ± 0.17 mm The inhibition zone for *P. aeruginosa* is 20 ± 0.21 mm When the extract is used in combination with commonly used antibiotics (imipenem, amikacin, ofloxacin), it shows a synergistic effect, with FICI values all less than 0.5	[85]
Tamarind fruit pulp	80% methanol extraction	In vitro (the microdilution method)	The extract exhibits varying degrees of antibacterial activity against clinically isolated *P. aeruginosa* and methicillin-resistant *S. aureus* (MRSA)	[86]
Tamarind leaves	Acetone and ethanol extracts	In vitro (disk diffusion method)	The antibacterial activity of ethanol extract > acetone extract The antibacterial activity of the extract against *K. pneumoniae* > *S. aureus*	[87]
Tamarind leaves	Distilled water and 95% ethanol cold soaking extraction	In vitro (agar Well Diffusion Method)	The antibacterial activity of ethanol extract > water extract Extract is more sensitive to *E. coli*	[88]
Tamarind leaves	Maceration and Soxhlet extractions	In vitro (total Viable Count Test)	Gentamicin is the standard drug, and the MBC and MIC of the extract for all tested strains are 500 μg/mL and 250 μg/mL The extract has the best inhibitory effect on *E. coli*	[89]
Prebiotic activity				
Tamarind seeds kernel	Dissolve in the culture medium	In vitro (drop plate method and crystal violet staining)	Bifidobacterium, biofilm formation ↑ Most significant at a concentration of 5%	[90]
Tamarind seeds	Polysaccharides from tamarind seeds (TSP)	In vitro (prebiotic Activity Test)	*Lactobacillus* ↑, *Salmonella* ↓	[91]
Tamarind seeds	TSP	In vitro (gastrointestinal digestion simulation experiment)	*Bifidobacterium*, *Parabacteroides*, and *Faecalibacterium* ↑ *Escherichia-Shigella* and *Dorea* ↓	[92]
Tamarind seeds	TSP	In vitro (gastrointestinal digestion simulation experiment)	*Escherichia-Shigella* and *Dorea* ↓ *Lactobacillus*, *Parabacteroides*, *Prevotella*, and *Faecalibacterium* ↑	[93]
Tamarind seeds	TSP	In vitro (Fermentation)	*Lactobacillus delbrueckii* and *Lactobacillus fermentum* ↑	[94]
Anti-inflammatory activity				
Tamarind seeds	Aqueous extract	In vivo (STZ-induced diabetes rats)	NO, TNF-α ↓	[95]
Tamarind seeds	95% ethanol extraction	In vivo (arthritis model)	ROS, H_2_O_2,_ LPO, PCC ↓ LPO, PCC ↑ Restore GSH, SOD, CAT, GST, GPx, GRdx,, ALT and AST	[96]
Tamarind seeds kernel	95% ethanol extraction	In vitro (RAW 264.7 cells)	Phagocytosis and NO production ↑ iNOS, IL-1β, IL-6, and TNF-α ↓ NF-κB and MAPKs pathways ↑	[48]
Tamarind leaves	Transfer the membrane gel loaded with extract	In vivo (carrageenan-induced rats)	The inhibition rates of paw edema volume at 1, 2, 3, and 4 h post-treatment were 25.32%, 59.09%, 76.03%, and 86.94%, respectively	[97]
Tamarind seeds	TS and *Curcuma longa* root extracts (ratio 2:1)	In vitro (carrageenan-induced)	TNF-α, IL-1β, iNOS and COX-2 ↓ The body weight-bearing capacity of the right hind limb ↑	[98]
Tamarind’s various organs	N-hexane extract	In vitro (RAW 264.7 macrophages)	NO ↓	[99]
Tamarind seeds	Treated with ethanol	In vivo (RAW 264.7 macrophages)	NO, TNF-α, IL-1β, and IL-6 ↓	[100]
Tamarind seeds	Trypsin inhibitor isolated from tamarind seeds (TTI)	In vivo (RAW 264.7 macrophages)	Concentrations of inflammatory cytokines in plasma ↓	[101]
Other activity				
Tamarind fruit pulp	70% ethanol extraction	In vivo (aluminum chloride induces rats)	TAO, AChE in brain and serum ↓ GPX, SOD ↑	[102]
Tamarind bark and seeds	Methanol extraction	In vitro (Ellman colorimetric method)	Anti Alzheimer: bark extract > seeds extract AChE, BuChE ↓	[103]
Tamarind fruit pulp and seeds	80% ethanol extraction	In vivo (3% acetic acid induction)	Degree of inflammation, extent of inflammation, glandular damage, and leukocyte infiltration ↓ MPO, MDA ↓ Anti ulcerative colitis: seeds > fruit pulp	[104]
Tamarind seeds coat	Methanol extraction	In vivo (induction of different ulcers)	Ulcer index ↓ The induced ulcer model shows the most potent anti-ulcer effect	[105]
Tamarind fruit pulp	Different extraction methods	In vitro (Plasmodium falciparum)	Chloroform extract showed moderate antimalarial activity (IC_50_ = 34.8 µ g/mL) The main component is 5-hydroxymethylfurfural	[23]
Tamarind leaves	Methanol extract	In vitro (plate diffusion method)	The anti-malaria activity is equivalent to the standard drug chloroquine Tartaric acid has anti-malaria activity	[106]
Tamarind leaves	70% ethanol extraction	In vitro (PLDH)	*P. falciparum* has the highest inhibitory activity IC_50_ is 5.9 ± 0.64 µ g/mL	[107]
Tamarind seeds	Trypsin inhibitor	In vitro	Showing good interaction with TMPRSS2 SARS-CoV-2 ↓	[108]
Tamarind stem bark	Anhydrous ethanol extraction	In vitro (KVD-NDV)	At a concentration of 250 mg/mL, no HA activity was observed Hemagglutinin, neuraminidase ↓	[109]
Tamarind seeds	-	In vitro (PLA2)	Quercetin exhibits optimal interaction with PLA2 Catechins, epicatechins, and apigenin also showed good binding affinity	[110]
Tamarind seeds	99.7% ethanol extraction	In vitro and in vivo	PLA2 ↓ is dose-dependent Thrombin, caseinase ↓	[111]
Tamarind seeds	95% ethanol extracts	In vitro (Ascaris suum)	Has significant insecticidal potential against *A. suum*	[112]
Tamarind leaves	95% ethanol extraction	In vitro and in vivo	The prolongation of QT and RR ↑ LDH, CPK ↓	[113]

Abbreviations: Nanotechnology encapsulated trypsin preparation, TTI; NMDA, N-Methyl-D-aspartate; SOD, superoxide dismutase; CAT, peroxidase; GSH, glutathione; ORAC, Oxygen Radical Absorbance Capacity; T-BHP, tert-Butyl Hydroperoxide; TAC, total antioxidant capacity; LDL, low-density lipoprotein; TG, triglyceride; HDL, high density lipoprotein; VLDL, very low density lipoprotein; HDL-C, high-density lipoprotein cholesterol; SCr, Serum Creatinine; SA, Serum Albumin; TP, Total Protein; Hb, Haemoglobin; RBCs, red blood cells; WBCs, white blood cells; BSA, bovine serum albumin; ROS, reactive oxygen species; H_2_O_2_, hydrogen peroxide; LPO, lipid peroxidation; PCC, protein carbonyl content; GPx, glutathione peroxidase; GRdx, glutathione reductase; ALT, alanine transaminase; AST, aspartate transaminase; TAO, oxidative stress; AChE, acetylcholinesterase; MPO, eroxidase; MDA, activity and malondialdehyde; PLDH, plasmodium lactate dehydrogenase; HA, Positive blood coagulation; PC, the positive control; TNF-α, tumor necrosis factor alpha; TTIP, the effect of the purified tamarind seeds trypsin inhibitor; ECW, the trypsin inhibitor isolated from tamarind seeds; NAFLD, Non-Alcoholic Fatty Liver Disease; TC, the total choles-terol; TGs, triglycerides; IC_50_, the half maximal inhibitory concentration; TMPRSS2, transmembrane serine protease 2; PLA2, phospholipase A2.

## Data Availability

No new data were created or analyzed in this study. Data sharing is not applicable to this article.
